# Deepening Sexual Desire and Erotic Fantasies Research in the ACE Spectrum: Comparing the Experiences of Asexual, Demisexual, Gray-Asexual, and Questioning People

**DOI:** 10.1007/s10508-023-02784-3

**Published:** 2024-01-11

**Authors:** Filippo Maria Nimbi, Caterina Appia, Annalisa Tanzilli, Guido Giovanardi, Vittorio Lingiardi

**Affiliations:** Department of Dynamic and Clinical Psychology and Health Studies, Sapienza University of Rome, Via Degli Apuli 1, 00185 Rome, Italy

**Keywords:** Asexuality, ACE spectrum, Sexual desire, Sexual fantasy, Sexual behavior

## Abstract

Over the past 30 years, an increasing number of people have identified within the asexual (ACE) spectrum recognizing an absence/low/situational sexual attraction to individuals of any gender. The current study aims to deepen the knowledge of sexual desire, erotic fantasies, and related emotions within the ACE spectrum. A total of 1072 Italian volunteers were recruited to take part to the present study via social media. Data were collected from October 2021 to January 2022 using the Sexual Desire and Erotic Fantasies questionnaire and the Sexual Desire Inventory-2. Participants were divided into four groups: asexual, demisexual, gray-asexual, and questioning. Focusing on sexual desire, asexual people reported significantly lower scores than the other groups in all the dimensions except for “negative feelings to sexual desire,” while demisexual participants showed the higher scores in all the domains except for “negative feelings to sexual desire.” The questioning group reported the highest scores in the “negative feelings toward sexual desire” compared to the asexual and demisexual ones. The asexual group reported significantly lower scores than the other groups in fantasies frequency, fantasies importance, negative emotions, and sharing and experiencing. The demisexual group showed higher frequency of romantic fantasies than the asexual and gray-asexual ones. The results showed some specific patterns of desire and fantasies among the asexual, gray-asexual, demisexual, and questioning groups. These data may provide relevant material to clinicians working with asexual patients who need greater awareness about the diversity and heterogeneity of the sexual experience within the ACE spectrum.

## Introduction

Over the past 30 years, an increasing number of people have identified themselves as “asexual” recognizing an absence of sexual attraction to individuals of any gender (Bogaert, 2004; Carrigan, 2011; Scherrer, 2008), as opposed to “allosexual” people who feel sexual attraction toward people of one or more genders (DeLuzio Chasin, 2011). Asexuality has intrigued clinicians and researchers because it challenges the assumption of sexual desire/attraction as a basic human drive and need (Brotto & Yule, 2017; Nimbi et al., 2020a, b). Consequently, on the one hand, some researchers have recognized asexuality as a heterogeneous entity that meets the criteria of a sexual orientation (Bogaert, 2006, 2012b, 2015; Brotto & Yule, 2017; Brotto et al., 2015), although there is not full agreement in the literature on this point. On the other hand, from an activist perspective, numerous actions have increased the recognition and visibility of the ACE spectrum (asexual or closely related sexual orientations as part of a broader community) among the LGBTQIA+ community (de Lappe, 2016).

Asexuality is often acknowledged as both an identity and an umbrella term encompassing closely related identities within a larger community. The asexual community displays significant diversity in sexual expression, relationships, attraction, and arousal. Researchers and clinicians are encouraged to recognize the diverse ways individuals may identify as asexual (Antonsen et al., 2020), even if their self-identification does not align completely with a specific definition of asexuality (Scherrer, 2008). Additionally, the terminology used to describe different identities within the asexual community, like demisexual or gray-asexual, is fluid, evolving, and deeply personal. To provide some basic definition to the reader, demisexual people do not feel sexual attraction unless they have established a strong emotional bond with partners, gray-asexual people can rarely feel sexual attraction, while questioning ones are exploring, not sure, or concerned about their asexual identity, and have not yet defined a more specific position in the ACE spectrum (Hille et al., 2020). The Ace Community Survey (Hermann et al., 2020) reported that, on 14.694 respondents from the international asexual community, 67.3% identified as asexual, 8.8% as demisexual, 10.5% as gray-asexual, and 11.2% as questioning.

Sexual experiences can encompass a wide range of elements varying from expressions, feelings, sensations, and solitary/partnered behaviors. Among these, a central place is occupied by sexual desire, i.e., a subjective psychological state for initiating and maintaining human sexual behavior, triggered by internal and/or external stimuli (Mark et al., 2014; Nimbi et al., 2020c). One of the expressions of desire is sexual fantasies, such as subjective mental images and thoughts that are erotic or arousing to the individual while awake (Leitenberg & Henning, 1995). These items represent the most frequent sexual experience in the allosexual population, with about 90–97 percent reporting experiencing erotic fantasies and using them to stimulate desire and arousal (Lehmiller, 2018; Nimbi et al., 2020c).

Contrary to stereotypes that portray asexual people as uninterested to sexual expressions or feelings, people who identify in the ACE spectrum do not necessarily abstain from sexual experiences (Nimbi et al., 2020a). The literature has shown a variability of approaches to sexuality, ranging from interest, revulsion, and indifference (often labelled as sex-positive, -negative and -neutral positions) (Bogaert, 2004; Carrigan, 2011; Doremus et al., 2023; Scherrer, 2008). Mitchell and Hunnicutt (2019) showed that many asexual individuals have engaged in sexual activities prior to embracing their asexual identity. Interestingly, the same study showed that all participants experienced relationships, bonding, and romance in unique and creative ways. Carrigan (2011) showed that some asexual people enjoyed arousal and orgasms in different ways than allosexual people. Asexual individuals were more likely to report never having had a sexual fantasy than others, although, in a more recent study (Skorska et al., 2023), sexual fantasies showed to be the preferred sexual stimuli to get aroused compared to porn. Often, erotic fantasies reported by asexual individuals do not involve other people. When present, they play a peripheral role or are faceless (Sloan, 2015; Yule et al., 2014, 2017). Some asexual individuals have reported that only objects, situations, or masturbation awaken desire and pleasure in them (Sloan, 2015). However, a more in-depth study of expressions of sexual desire and erotic fantasies is lacking in the literature (Nimbi et al., 2020a, b), especially regarding the different identities that are part of the ACE spectrum such as demisexual and gray-asexual people, which have never been subjected to research except for few exceptions. Furthermore, little is known about people who define themselves as questioning (Copulsky & Hammack, 2023; Hille et al., 2020).

An in-depth study of the different expressions of sexual desire and fantasies by making a comparison between the main identity groups within the ACE spectrum could be useful not only to deepen a knowledge gap for itself. In fact, recognizing whether different identities exhibit similar patterns in the expression of desire and sexual fantasies can help asexual people in their process of self-identification, posing terms of comparison based on recognizable elements that are in line with their self-representation. In addition, the recognition of such patterns leads to more evidence that asexuality is not a desire disorder (due to the absence of a personal distress) (American Psychiatric Association, 2013; World Health Organization, 2019). Thus clinicians may become more familiar with the characteristics of the ACE population, being more sensitive to individual experiences and less judgmental toward sexual diversities (Nimbi et al., 2021).

### Aims

The current study aimed to deepen the knowledge of sexual desire, erotic fantasies, and related emotions within the ACE spectrum. Drawing on a sex-positive approach (Nimbi et al., 2021) as a reference, different sexual expressions are understood as natural part of individual variability and not as expressions of dysfunction or pathology.

The first aim of the present study is to compare different sexual desire expressions (e.g., object, contexts, emotions) and erotic fantasies (attitudes and content) among four groups within the ACE spectrum: asexual, demisexual, gray-asexual, and questioning individuals. Secondly, the study aimed to explore some characteristics associated to the most commonly reported erotic fantasies within these four groups. We expect to find varying expressions of desire and related emotions that align with the self-definition of participants’ sexual identity.

## Method

### Participants

A total of 1072 Italian volunteers were recruited to take part to the present study via social media (Instagram, Facebook, and LinkedIn) on profiles related to asexual/ACE awareness, activism, and ACE spectrum collectives/associations. Data were collected using Google.forms from October 2021 to January 2022. All participants were asked to complete an informed consent form before accessing the survey. The questionnaire administered was anonymous, and no remuneration was provided to participants. The questionnaire took approximately 30 min to be completed.

Inclusion criteria were being fluent in Italian language, being 18 years old or above, and declaring to self-identifying in the ACE spectrum. Control questions were included in the survey to recognize and eliminate falsified answers. According to these criteria, data from 31 participants (2.89% of the total sample) were excluded from the study, because they declared to be younger and/or to not identify in the ACE spectrum and/or were detected as falsified records. The final group resulted in 1041 participants (Table 1).

**Table 1:** Sociodemographic data and description of the participants (*n* = 1041)

	Total group (*n* = 1041)	Asexual (*A*) (*n* = 297)	Demisexual (*D*) (*n* = 331)	Gray-Asexual (*G*) (*n* = 124)	Questioning (*Q*) (*n* = 289)	Significance
	*M* ± SD	*M* ± SD	*M* ± SD	*M* ± SD	*M* ± SD	*F*(3, 1037)
Age	25.25 ± 5.7	25.22 ± 6.77	25.85 ± 5.53	24.49 ± 4.79	24.9 ± 4.98	*p* = 0.072
Gender	*n* (%)	*n* (%)	*n* (%)	*n* (%)	*n* (%)	*χ*^2^
Female	718 (68.97)	175 (58.92)	247 (74.62)	68 (54.83)	228 (78.89)	56.9 df = 9 *p* < 0.001
Male	81 (7.78)	24 (8.08)	23 (6.94)	18 (14.51)	16 (5.53)	
Questioning	89 (8.54)	30 (10.1)	23 (6.94)	11 (8.87)	25 (8.65)	
Non-binary	153 (14.69)	68 (22.89)	38 (11.48)	27 (21.77)	20 (6.92)	
Sex assigned at birth						
Female	952 (91.45)	270 (90.9)	311 (93.95)	104 (83.87)	267 (92.38)	15.1 df = 6 *p* = 0.02
Male	88 (8.45)	27 (9.76)	19 (5.74)	20 (16.12)	22 (7.61)	
Other	1 (0.09)	0	1 (0.3)	0	0	
Education degree						
Middle school	35 (3.36)	11 (3.7)	8 (2.41)	5 (4.03)	11 (3.8)	16.99 df = 9 *p* = 0.049
High school	525 (50.43)	173 (58.24)	150 (45.31)	66 (53.22)	136 (47.05)	
Degree	381 (36.59)	94 (31.64)	133 (40.18)	43 (34.67)	111 (38.4)	
Post-degree	100 (9.6)	19 (6.39)	40 (12.08)	10 (8.06)	31 (10.72)	
Work status						
Unemployed	91 (8.74)	33 (11.11)	30 (9.06)	8 (6.45)	20 (6.92)	*p* = 0.054
Employed	272 (26.12)	79 (26.59)	94 (28.39)	19 (15.32)	80 (27.68)	
Freelance	85 (8.16)	17 (5.72)	30 (9.06)	14 (11.29)	24 (8.3)	
Student	591 (56.77)	167 (56.22)	177 (53.47)	82 (66.12)	165 (57.09)	
Retired	2 (0.19)	1 (0.33)	0	1 (.8)	0	
Political orientation						
Extreme-Left	268 (25.74)	82 (27.6)	83 (25.07)	32 (25.8)	71 (24.56)	*p* = 0.3
Moderate-Left	619 (59.46)	155 (52.18)	212 (64.04)	75 (60.48)	177 (61.24)	
Center	44 (4.22)	18 (6.06)	11 (3.32)	6 (4.83)	9 (3.11)	
Moderate-Right	19 (1.82)	7 (2.35)	2 (0.6)	4 (3.22)	6 (2.07)	
Extreme-Right	2 (0.19)	1 (0.33)	1 (0.3)	0	0	
Other parties	89 (8.54)	21 (7.07)	30 (9.06)	16 (12.9)	22 (7.61)	
Religion						
Atheist/Agnostic	825 (79.25)	236 (79.46)	242 (73.11)	107 (86.29)	240 (83.04)	*p* = 0.076
Christian	149 (14.31)	40 (13.46)	81 (24.47)	11 (8.87)	37 (12.8)	
Buddhist	11 (1.05)	1 (0.33)	7 (2.11)	0	3 (1.03)	
Jewish	2 (0.19)	1 (0.33)	1 (0.3)	0	0	
Islam	2 (0.19)	1 (0.33)	0	0	1 (0.34)	
Others	52 (4.99)	18 (6.06)	20 (6.04)	6 (4.83)	8 (2.76)	
Romantic orientation						
Aromantic	76 (7.3)	54 (18.18)	3 (0.9)	12 (9.67)	7 (2.42)	114.66 df = 12 *p* < 0.001
Homoromantic	100 (9.6)	10 (3.36)	40 (12.08)	15 (12.09)	35 (12.11)	
Heteroromantic	297 (28.53)	69 (23.23)	109 (32.93)	34 (27.41)	85 (29.41)	
Q uestioning	200 (19.21)	72 (24.24)	49 (14.8)	15 (12.09)	64 (22.14)	
Bi/Panromantic	368 (35.35)	92 (30.97)	130 (39.27)	48 (38.7)	98 (33.91)	
Current relational status						
Single	623 (59.84)	213 (71.71)	174 (52.56)	70 (56.45)	166 (57.43)	29.69 df = 9 *p* < 0.001
Monogamous couple	381 (36.59)	75 (25.25)	144 (43.5)	47 (37.9)	115 (39.79)	
Polyamorous/non-monogamous relationship	34 (3.26)	8 (2.69)	12 (3.62)	7 (5.64)	7 (2.42)	
Queer platonic relationship	3 (0.28)	1 (0.33)	1 (0.3)	0	1 (0.34)	
Sexual intercourses in life						
No	340 (32.66)	168 (56.56)	67 (20.24)	32 (25.8)	73 (25.25)	110.23 df = 3 *p* < 0.001
Yes	701 (67.33)	129 (43.43)	264 (79.75)	92 (74.19)	216 (74.74)	
Sexual intercourses last six months						
No	576 (55.33)	224 (75.42)	154 (46.52)	59 (47.58)	139 (48.09)	68.02 df = 3 *p* < 0.001
Yes	465 (44.66)	73 (24.57)	177 (53.47)	65 (52.41)	150 (51.9)	

	*M* ± SD	*M* ± SD	*M* ± SD	*M* ± SD	*M* ± SD	*F* (3, 1037)
Sexual activity frequency in the last six months	2.15 ± 1.45	1.62 ± 1.13	2.51 ± 1.59	2.21 ± 1.43	2.26 ± 1.42	21.99 *p* < 0.001 *A* < *D*, *G*, *Q*
Masturbation frequency in the last six months	3.39 ± 1.58	3.23 ± 1.61	3.47 ± 1.53	3.38 ± 1.62	3.47 ± 1.54	*p* = 0.197
Porn consumption frequency in the last six months	2.64 ± 1.5	2.44 ± 1.48	2.66 ± 1.41	2.76 ± 1.6	2.75 ± 1.53	*p* = 0.05

### Measures

Participants completed a web survey composed by an ad hoc form collecting sociodemographic information (such as age, gender, sexual and romantic orientation, marital and relational status, being sexually active, education level, work status, religious, and political orientation) and five validated measures:

The Sexual Desire Inventory-2 (SDI-2; Spector et al., 1996) is a 14-item measure which evaluates the dyadic and solitary dimensions of sexual desire. Spector et al. defined sexual desire as an interest in sexual activity, measured by the quantity and strength of thought invested in sexual stimuli. This questionnaire has frequently been used in literature in the last decades as a quick and easy measure to administer. The two-dimensional structure presents satisfying psychometric properties also in the Italian version (Callea & Rossi, 2021). Both dimensions were calculated as a sum of the corresponding items, where higher scores indicate a higher level of sexual desire. In the current study, dyadic desire dimension scores ranged from 0 to 64 and solitary desire ranged from 0 to 31. The Cronbach’s alpha values for this measure in the current study ranged from 0.91 (dyadic desire) to 0.93 (solitary desire).

The Sexual Desire and Erotic Fantasies questionnaire (SDEF; Nimbi et al., 2023a, b, c) is composed of three stand-alone questionnaires (1. Sexual Desire; 2. Use of Erotic Fantasies; and 3. Erotic Fantasies Inventory) that may be assessed together for a general overview of desire functioning or separated with different purposes. These questionnaires have been validated in the Italian population (Nimbi et al., 2023a, b, c) and are conceptualized with the specific aim of providing a tool for measuring sexual desire and fantasies based on a sex positive-approach (Nimbi et al., 2021), thus inclusive toward a broad range of sexual identities, such as the ACE spectrum.

The SDEF1 (Nimbi et al., 2023a) is a 28-item questionnaire measuring six aspects related to sexual desire: sexual desire, negative feelings to sexual desire, autoerotic desire, regular partner desire, (self-defined) attractive person desire, and responsive desire. The answers were expressed on 5- and 6-step Likert scales (ranging from “Never” to “More times per day/Always”). Higher scores indicate a higher level of sexual desires/feelings. In the current study, the scores ranged 0–41 for sexual desire, 0–25 for negative feelings to sexual desire, 0–15 for autoerotic desire, regular partner desire, attractive person desire, and responsive desire. Some items presented unscored solutions indicated with a hash mark to express the inability to answer the question for a specific reason (e.g., “#. I don't have a regular partner, or I have never had desire for a regular partner”). The Cronbach’s alpha values for this measure in the current study ranged from 0.57 (attractive person desire) to 0.86 (sexual desire). We specify that the low level of alpha in respect of the desire for an attractive person scale is motivated by the high number of participants who did not respond to some related items, in line with the definition of the ACE spectrum in which there is a reduced/lack of sexual attraction.

The SDEF2 (Nimbi et al., 2023a) is a 21-item questionnaire exploring five domains related to erotic fantasies attitudes and use: fantasies frequency, fantasies normality, importance given to fantasies, negative emotions related to the experience of erotic fantasies, and sharing and experiences of erotic fantasies with regular partners. Participants expressed their agreement or the intensity of their experience on 5- and 6-step Likert scales (ranging from “Never” to “More times per day/Always”). Some items have unscored solutions indicated with an asterisk to express the inability to answer the question for a specific reason (e.g., “#. I have never had erotic fantasies”). Higher scores indicate a higher frequency of sexual fantasies/accordance with the items. In the current study, the scores ranged 0–22 for fantasies frequency, 0–12 for fantasies normality and importance given to fantasies, 0–24 for negative emotions related to the experience of erotic fantasies, and 0–14 for sharing and experiences of erotic fantasies with regular partners. The Cronbach’s alpha values for this measure in the current study ranged from 0.72 (fantasies frequency) to 0.91 (importance given to fantasies).

The SDEF3 (Nimbi et al., 2023b) is a 125-item questionnaire assessing the frequency of the most common erotic fantasies based on an inclusive and updated list of erotic situations/practices/objects. Answers were rated using a 5-point Likert scale (from “never” to “always”) to indicate the frequency of erotic fantasies related to the presented stimuli in the last six months. All domain scores were computed as mean, ranging from 0 to 4 where higher scores indicate a higher frequency of fantasies. At the end of the questionnaire, a part is left free for the person to indicate fantasies that have not been included in the previous items as open ended questions. The SDEF3 has 2-dimensional structures: A 6-dimension factorial structure providing general categories of erotic fantasies (Physical and contextual, BDSM, Taboo, Bottom, Top, and Romantic) that showed good psychometric characteristics, and an extended version composed of 20 dimensions, suggested for clinical and explorative/descriptive contexts. The Cronbach’s alpha values for this measure in the current study ranged from 0.79 (taboo) to 0.91 (BDSM).

To clarify the dimensions of sexual desire assessed in this study, a brief description of each variable is reported in Table 2.

**Table 2:** Description of the dimensions of the Sexual Desire Inventory-2 and the Sexual Desire and Erotic Fantasies Questionnaire

Questionnaire	Dimensions	Description
Sexual Desire Inventory-2 (SDI-2) 2-dimension factorial structure	F1. Dyadic desire (9 items)	Dimension that measures desire within the couple's relationship
	F2. Solitary desire (4 items)	Dimension that measures the desire for masturbation and solo sexual activity
Sexual Desire and Erotic Fantasies Questionnaire – 1. Sexual Desire (SDEF 1) 6-Dimension factorial structure	F1. Sexual Desire (9 items)	This dimension delineates the individual's perceived intensity of sexual desire by examining activities including kissing, manual/oral stimulations, and penetrative intercourses
	F2. Negative Feelings to Sexual Desire (7 items)	Within this overarching dimension, distressing and adverse emotions associated with sexual desire, coupled with endeavors to diminish or regulate desire and their ensuing negative repercussions, are comprehensively assessed
	F3. Autoerotic Desire (3 items)	This dimension characterizes the longing for and contentment derived from individual sexual activities, such as masturbation
	F4. Regular Partner Desire (3 items)	Within this dimension, the desire for and contentment derived from sexual activities with a consistent partner are explored. This encompasses committed relationships with steady partners as well as enduring connections like friends-with-benefits
	F5. Attractive Person Desire (3 items)	This dimension delineates the inclination and contentment associated with engaging in sexual activities with an appealing individual. It is emphasized that this person should differ from the current regular partner (if any), as F5 aims to gauge desire for a liked stimulus
	F6. Responsive Desire (3 items)	This dimension explores desire as the openness to a partner's sexual advances and seduction. Grounded in the concept of “responsive sexual desire,” it reflects a scenario where individuals willingly participate in sexual activity, despite an initial absence of desire or arousal. Through adequate sexual stimuli and within suitable contexts, an individual can transition from a neutral state to experiencing arousal and desire
Sexual Desire and Erotic Fantasies Questionnaire – 2. Use of Erotic Fantasies (SDEF 2) 5-Dimension factorial structure	F1. Fantasies Frequency (5 items)	This dimension details the self-reported occurrence of erotic fantasies across various sexual and non-sexual contexts
	F2. Fantasies Normality (3 items)	Within this dimension, the individual's perception of the normalcy of having erotic fantasies is examined, encompassing general situations, masturbation, and sexual activity with a partner
	F3. Fantasies Importance (3 items)	This dimension gathers insights into the significance and functional role attributed to erotic fantasies in the sexual experience
	F4. Negative Emotions (6 items)	This dimension compiles a spectrum of adverse emotional responses to erotic fantasies, including discomfort, worry, a sense of guilt, anger, frustration, and embarrassment
	F5. Sharing and Experiencing (4 items)	This dimension describes how frequently an individual shares and engages in experimentation with their erotic fantasies, particularly with a regular partner (if any)
Sexual Desire and Erotic Fantasies Questionnaire – 3. Erotic Fantasies Inventory (SDEF 3) 6-Dimension factorial structure	F1. Physical and Contextual (19 items)	An array of physical attributes influenced by widely embraced cultural standards of beauty, such as an athletic or slender physique, tall stature, and youthful appearance. This also includes settings or scenarios deemed erotically arousing and emblematic in mainstream pornography, such as outdoor intimacy, seduction, and workplace encounters
	F2. BDSM (13 items)	Various sexual scenarios evoking elements of BDSM and bondage practices, engaging in sadomasochistic activities, exploring fetishism, and related themes
	F3. Taboo (12 items)	This domain involves taboo fantasies such as having sexual intercourses with animals, children, relatives, corpses, and rape among other themes
	F4. Bottom (8 items)	This encompasses a variety of common sexual activities wherein an individual assumes the role of receiving the practice (bottom/passive), while their partner takes on a more leading and active role
	F5. Top (7 items)	This involves a spectrum of common sexual activities where the person takes on the role of performing the practice (leader/active), with their partner adopting a more passive or bottom role in receiving it
	F6. Romantic (7 items)	Delving into romantic interactions, this includes scenarios and gestures like kissing, hugging, massage, and mutual gazes, with partners alternately looking after or being looked after
Sexual Desire and Erotic Fantasies Questionnaire – 3. Erotic Fantasies Inventory (SDEF 3) 20-Dimension factorial structure	F1. Physical Characteristics	This dimension collects erotic fantasies based on physical characteristics culturally attributed to being beautiful/handsome, attractive, and sexy such as athletic/thin body, young age, etc.
	F2. Group sex	Dimension describing sexual scenarios having more than one sexual partner involved
	F3. Romantic	Romantic scenarios and interactions, this includes gestures like kissing, hugging, massage, and mutual gazes, with partners alternately looking after or being looked after
	F4. Vanilla Sex	Range of common sexual practices ranging from petting, oral sex, masturbation to vaginal sex
	F5. Masochism	Torture, humiliating, and painful practices that are arousing for the participant who received them
	F6. Sadism	Torture, humiliating, and painful practices that are arousing for the participant who perform them
	F7. Taboo	Taboo fantasies such as having sexual intercourses with animals, children, relatives, and corpses
	F8. Anal Sex and Toys	Activities involving anal play and sex toys
	F9. Incestuous/Older people	Range of fantasies about family members, elderly people, pregnant women, and obese people
	F10. Soft Fetish	Range of fetishes that includes foot, hair, saliva, sweat, and other parts of the body
	F11. Risk of Being Caught	Fantasies characterized by open air scenarios or places in which is easy to be caught while having sex
	F12. Past Experience	Memories of past sexual experiences and former partners
	F13. Seduction and Infidelity	Fantasies in which seduction and betrayal of a relationship are central
	F14. Exhibitionism and Voyeurism	Fantasies in which watching or being spied on while naked or engaging in sexual activity are central
	F15. Bondage	Range of practices involving the action of tying/being tied up and blindfolded
	F16.Sexual Abuse	Erotic fantasies about non-consensual sexual activities
	F17. Sex work	Scenarios in which the sexual activity is bought or sold, including playing in a porn movie
	F18. Ejaculation Emission	Fantasies focused on the act of ejaculating on the partner
	F19. Receiving Ejaculation	Fantasies focused on the act receiving the ejaculation of the partner
	F20. Dirty Fetish	Fetish for liquids such as urine, excrement, and vomit

The Marlow-Crowne Social Desirability Scale-Short Form (MCSDS-SF; Fischer & Fick, 1993), a 13-item measure of socially desirable responses. The respondent indicates how true or false the presented statement is. Each true answer awards one point, false zero. Higher scores indicate a higher tendency to respond in a more socially desirable way. The MCSDS–SF was used as a covariate in the analysis of the current study to limit the effects of social desirability. The Cronbach’s alpha values for this measure in the current study were 0.93.

### Data Analysis

First, sociodemographic data were discussed to highlight the characteristics of the groups. Participants were then divided into four groups according to their declared sexual orientation: asexual, demisexual, gray-asexual, and questioning. Five multivariate analyses of covariance (MANCOVA) were conducted to test differences between sexual orientations mean scores at the SDI-2 and SDEF subdimensions having the MCSDS-SF total score as a covariate. Post hoc analyses using the Bonferroni method were conducted with multiple comparisons to highlight the differences among sexual orientation groups. Data analysis was carried out using the IBM SPSS Statistic software v. 27.00.

## Results

Participants consisted of 1041 people declaring to be part of the ACE spectrum, whose mean age was 25.25 ± 5.71 years, ranging from 18 to 57 years old. Sociodemographic data for the total group and the subgroups (asexual, demisexual, gray-asexual, and questioning) are summarized in Table 1. Most participants were cisgender women and non-binary people who were assigned female at birth, have a middle-high level of education, and were students or employed. Most of the group were politically left-winged and reported to be atheist/agnostic. Regarding romantic orientation, most of the group reported to be bi/panromantic and heteroromantic, to be single or in a monogamous relationship. In particular, the asexual group was more likely to report an aromantic orientation and to be single. Regarding sexual experiences, most of the participants reported to have had at least a sexual intercourse in their life, while more than half declared to not have had sex in the 6 months prior to the study assessment, with the asexual group reporting less sexual intercourses than the other groups. The asexual group reported to have less frequent partnered sexual activity than all the other groups, while no significant difference emerged on masturbation and porn consumption among the groups.

Focusing on sexual desire assessed with the SDI-2, Table 3 shows the results of a MANCOVA reporting a significant difference among the sexual orientation groups having social desirability as covariate [*F*(3, 1036) = 57.26; *p* < 0.001; Wilk's Λ = 0.736; partial *η*^2^ = 0.142].The Bonferroni postdoc analyses showed that the asexual group had significantly lower scores than the other groups in solitary and dyadic desire, while the gray-asexual group reported significantly lower scores than the demisexual and questioning ones in dyadic desire.

**Table 3:** MANCOVAs among sexual orientation groups having social desirability as covariate (*n* = 1041)

	Asexual (*A*) (*n* = 297)	Demisexual (*D*) (*n* = 331)	Grey-Asexual (*G*) (*n* = 124)	Questioning (*Q*) (*n* = 289)	Post hoc Bonferroni	*F*(3, 1037)	*p*	Partial Eta^2^
	*M* ± SD	*M* ± SD	*M* ± SD	*M* ± SD				
SDI-2 domains								
Solitary desire	11.17 ± 8.51	13.2 ± 8.29	12.73 ± 8.63	14.38 ± 8.6	*A* < *D*, *Q*	7.15	< 0.001	0.02
Dyadic desire	9.07 ± 9.65	26.77 ± 12.9	19.27 ± 12.29	24.27 ± 14.5	*A* < *G* < *D*, Q	120.66	< 0.001	0.259
SDEF 1 domains								
Sexual desire	8.48 ± 5.72	15.37 ± 7.73	12.57 ± 6.81	13.5 ± 7.37	*A* < *G*, *Q* < *D*	54.06	< 0.001	0.135
Negative feelings to sexual desire	4.29 ± 4.17	4.90 ± 4.17	5.02 ± 3.95	5.89 ± 4.37	*Q* > *A*, *D*	6.95	< 0.001	0.02
Autoerotic desire	4.89 ± 3.53	5.69 ± 3.27	5.02 ± 3.95	5.98 ± 3.36	*A* < *D*, *Q*	5.51	.001	0.016
Regular partner desire	1.19 ± 2.33	3.80 ± 3.88	2.41 ± 2.93	2.63 ± 3.19	*A* < *G*, *Q* < *D*	35.54	< 0.001	0.093
Attractive person desire	0.93 ± 1.64	2.14 ± 2.26	2.23 ± 2.24	2.59 ± 2.54	*A* < *D*, *G*, *Q*	31.14	< 0.001	0.083
Responsive desire	5.26 ± 1.71	7.09 ± 2.59	6.05 ± 2.16	6.32 ± 2.47	*A* < *G*, *Q* < *D*	34.09	< 0.001	0.09
SDEF 2 domains								
Fantasies frequency	5.64 ± 3.91	7.34 ± 4	6.81 ± 3.88	7.6 ± 4.12	*A* < *D*, *G*, *Q*	25.5	< 0.001	0.024
Fantasies normality	9.29 ± 2.45	9.73 ± 2.05	9.51 ± 2.11	10.11 ± 1.92	–	–	0.057	–
Fantasies importance	6.38 ± 3.45	7.07 ± 3.01	6.65 ± 3.31	7.78 ± 3.03	*A* < *D*, *Q* *Q* > *A*, *D*, *G*	13.17	< 0.001	0.013
Negative emotions	3.5 ± 4.75	4.2 ± 4.61	4.91 ± 5.29	5.16 ± 5.37	*A* < *G*, *Q*	15.99	< 0.001	0.015
Sharing and experiencing	1.13 ± 2.47	2.73 ± 3.4	2.06 ± 2.83	2 ± 2.93	*A* < *D*, *G*, *Q* *Q* < *D*	9.08	0.003	0.009
SDEF 3 domains								
Physical and contextual	0.43 ± 0.52	0.71 ± 0.57	0.73 ± 0.63	0.87 ± 0.61	*A* < *D*, *G*, *Q* *Q* > *A*, *D*	28.94	< 0.001	0.077
BDSM	0.37 ± 0.56	0.65 ± 0.72	0.57 ± 0.66	0.59 ± 0.73	*A* < *D*, *G*, *Q*	10.27	< 0.001	0.029
Taboo	0.07 ± 0.21	0.05 ± 0.12	0.08 ± 0.17	0.08 ± 0.18	–	–	0.084	–
Bottom	0.42 ± 0.65	0.85 ± 0.78	0.69 ± 0.64	0.79 ± 0.75	*A* < *D*, *G*, *Q*	22.12	< 0.001	0.06
Top	0.34 ± 0.54	0.69 ± 0.73	0.57 ± 0.73	0.61 ± 0.68	*A* < *D*, *G*, *Q*	15.47	< 0.001	0.043
Romantic	1.07 ± 0.89	1.64 ± 0.98	1.37 ± 0.88	1.45 ± 0.91	*A* < *D*, *G*, *Q* *D* > *A*, *G*	20.32	< 0.001	0.056

Regarding sexual desire dimensions assessed with the SDEF1, Table 3 shows the results of a MANCOVA with a significant difference among sexual orientations having social desirability as covariate (Wilk's Λ = 0.796; *p* < 0.001; partial *η*^2^ = 0.073). The Bonferroni postdoc analyses showed that asexual people have significantly lower scores than the other groups in all of the dimensions except for “negative feelings to sexual desire,” while demisexual participants showed the higher scores in all the domains assessed by the SDEF1 (except for “negative feelings to sexual desire”). The questioning group reported the highest scores in the “negative feelings toward sexual desire” compared to asexual and demisexual ones.

Focusing on erotic fantasies use and attitudes (assessed with the SDEF2), Table 3 shows the results of a MANCOVA reporting a significant difference among the sexual orientation groups having social desirability as covariate (Wilk's Λ = 0.903; *p* < 0.001; partial *η*^2^ = 0.033).The Bonferroni postdoc analyses showed that the asexual group reached significantly lower scores than the other groups in fantasies frequency, fantasies importance, negative emotions, and sharing and experiencing. The questioning group showed significant higher score than the other groups in the dimension expressing the importance attributed to fantasies.

Regarding erotic fantasies topics assessed with the SDEF3, Table 3 shows the results of a MANCOVA with a significant difference among sexual orientations having social desirability as covariate (Wilk's Λ = 0.846; *p* < 0.001; partial *η*^2^ = 0.054). The Bonferroni postdoc analyses showed that asexual people have significantly lower scores than the other groups in all of the areas except for taboo fantasies. Questioning participants reported higher scores on physical and contextual fantasies than asexual and demisexual ones. The demisexual group showed higher frequency of romantic fantasies than asexual and gray-asexual ones.

To get a deeper insight into the content of erotic fantasies, the 20-factor version of the SDEF3 was computed and analyzed. Table 4 shows the results of a MANCOVA with a significant difference among sexual orientations having social desirability as covariate (Wilk's Λ = 0.774; *p* < 0.001; partial *η*^2^ = 0.082) in all of the areas except for sexual abuse and dirty fetish fantasies. The Bonferroni postdoc analyses showed that asexual people have significantly lower scores than the other groups in most of the categories. Moreover, difference highlighted in romantic, vanilla sex, risk of being caught, and seduction and infidelity fantasies showed a medium effect size.

**Table 4:** MANCOVA among sexual orientation groups having social desirability as covariate (*n* = 1041)

SDEF 3 domains (extended version)	Asexual (*A*) (*n* = 297)	Demisexual (*D*) (*n* = 331)	Grey-Asexual (*G*) (*n* = 124)	Questioning (*Q*) (*n* = 289)	Post Hoc Bonferroni	*F*(3, 1037)	*p*	Partial Eta^2^
	*M* ± SD	*M* ± SD	*M* ± SD	*M* ± SD				
F1. Physical Characteristics	0.27 ± 0.45	0.38 ± 0.52	0.47 ± 0.58	0.51 ± 0.57	*A* < *G*, *Q* *D* < *Q*	10.71	< 0.001	0.03
F2. Group Sex	0.29 ± 0.53	0.47 ± 0.66	0.47 ± 0.57	0.6 ± 0.74	*A* < *D*, *G*, *Q*	11.52	< 0.001	0.032
F3. Romantic	1.08 ± 0.89	1.64 ± 0.98	1.37 ± 0.88	1.45 ± 0.91	*A* < *D*, *G*, *Q* *G* < *D*	20.32	< 0.001	0.056
F4. Vanilla Sex	0.75 ± 0.88	1.55 ± 1	1.18 ± 0.88	1.46 ± 0.96	*A* < *G* < *D*, *Q*	44.54	< 0.001	0.114
F5. Masochism	0.42 ± 0.76	0.62 ± 0.77	0.59 ± 0.76	0.6 ± 0.79	*A* < *D*, *Q*	4.29	0.005	0.012
F6. Sadism	0.21 ± 0.49	0.4 ± 0.65	0.4 ± 0.67	0.38 ± 0.7	*A* < *D*, *G*, *Q*	6.02	< 0.001	0.017
F7. Taboo	0.06 ± 0.21	0.06 ± 0.16	0.05 ± 0.13	0.1 ± 0.22	*D* < *Q*	3.21	0.022	0.009
F8. Anal Sex and Toys	0.4 ± 0.64	0.69 ± 0.85	0.61 ± 0.8	0.64 ± 0.78	*A* < *D*, *Q *	8.49	< 0.001	0.024
F9. Incestuous/Older people	0.17 ± 0.36	0.16 ± 0.27	0.25 ± 0.38	0.28 ± 0.4	*A*, *D* < *Q*	7.21	< 0.001	0.02
F10. Soft Fetish	0.18 ± 0.38	0.32 ± 0.49	0.26 ± 0.49	0.33 ± 0.51	*A* < *D*, *Q*	7.11	< 0.001	0.02
F11. Risk of being Caught	0.36 ± 0.69	0.85 ± 0.98	0.59 ± 0.87	0.83 ± 0.97	*A* < *D*, *Q* *G* < *D*	20.49	< 0.001	0.056
F12. Past Experience	0.48 ± 0.61	0.87 ± 0.81	0.81 ± 0.78	0.85 ± 0.78	*A* < *D*, *G*, *Q*	17.83	< 0.001	0.049
F13. Seduction and Infidelity	0.63 ± 0.78	1.05 ± 0.85	1.08 ± 0.92	1.29 ± 0.87	*A* < *D*, *G*, *Q* *D* < *Q*	30.37	< 0.001	0.081
F14. Exhibitionism and Voyeurism	0.26 ± 0.56	0.37 ± 0.59	0.32 ± 0.58	0.42 ± 0.69	*A* < *Q*	3.55	0.014	0.01
F15. Bondage	0.47 ± 0.77	0.84 ± 0.98	0.63 ± 0.82	0.74 ± 0.91	*A* < *D*, *Q*	10.09	< 0.001	0.028
F16. Sexual Abuse	0.11 ± 0.4	0.07 ± 0.25	0.12 ± 0.4	0.08 ± 0.26	–	–	0.349	–
F17. Sex Work	0.12 ± 0.36	0.15 ± 0.39	0.15 ± 0.34	0.23 ± 0.51	*A* < *Q*	3.18	0.023	0.009
F18. Ejaculation Emission	0.16 ± 0.51	0.36 ± 0.83	0.38 ± 0.82	0.31 ± 0.78	*A* < *D*, *G*	4.9	0.002	0.014
F19. Receiving Ejaculation	0.28 ± 0.69	0.75 ± 1.04	0.58 ± 0.84	0.63 ± 1.02	*A* < *D*, *G*, *Q*	14.62	< 0.001	0.041
F20. Dirty Fetish	0.06 ± 0.24	0.06 ± 0.22	0.08 ± 0.33	0.06 ± .23	–	–	0.847	–

To have a qualitative look of the fantasies’ topic, Table 5 shows the most frequent fantasies reported by the total group and the four sub-groups based on SDEF3 items. “Caressing and hugging (cuddling)” and “Kissing a partner” are the two patterns that hold the highest places in the ranking in all groups. From the third position onward, a diversification by group can be observed.

**Table 5:** Most frequent erotic fantasies sorted by total group ranking (*n* = 1041)

Sexual Fantasy SDEF 3 items	Total Group (*n* = 1041)	Asexual (*A*) (*n* = 297)	Demisexual (*D*) (*n* = 331)	Grey-Asexual (*G*) (*n* = 124)	Questioning (*Q*) (*n* = 289)
	*M* ± SD (ranking)	*M* ± SD (ranking)	*M* ± SD (ranking)	*M* ± SD (ranking)	*M* ± SD (ranking)
3. Caressing and hugging (cuddling)	2.14 ± 1.38 (1)	1.77 ± 1.4 (1)	2.4 ± 1.39 (1)	2.08 ± 1.27 (1)	2.26 ± 1.31 (1)
2. Kissing a partner	2.04 ± 1.34 (2)	1.53 ± 1.36 (2)	2.32 ± 1.32 (2)	2 ± 1.16 (2)	2.26 ± 1.26 (2)
87. Engaging in sexual activity with a man	1.51 ± 1.36 (3)	1.05 ± 1.27 (5)	1.66 ± 1.37 (6)	1.57 ± 1.25 (3)	1.79 ± 1.35 (3)
86. Engaging in sexual activity with a woman	1.42 ± 1.37 (4)	0.9 ± 1.18 (8)	1.57 ± 1.4 (10)	1.51 ± 1.31 (4)	1.73 ± 1.37 (4)
7. Being masturbated by a partner	1.38 ± 1.31 (5)	0.81 ± 1.16 (13)	1.7 ± 1.29 (4)	1.33 ± 1.22 (8)	1.63 ± 1.32 (5)
12. Having vaginal intercourse with a receptive role (bottom/passive; with penis or sex toys)	1.36 ± 1.37 (6)	0.87 ± 1.2 (11)	1.65 ± 1.4 (7)	1.25 ± 1.28 (12)	1.59 ± 1.4 (8)
26. Letting yourself be looked after by the partner	1.34 ± 1.33 (7)	1.13 ± 1.25 (3)	1.56 ± 1.39 (10)	1.37 ± 1.31 (6)	1.28 ± 1.34 (17)
6. Touching the breast/chest or stimulating the nipples	1.33 ± 1.33 (8)	0.9 ± 1.21 (9)	1.53 ± 1.33 (11)	1.323 ± 1.25 (9)	1.55 ± 1.36 (10)
10. Receiving oral sex (cunnilingus/fellatio/anilingus)	1.32 ± 1.33 (9)	0.74 ± 1.23 (17)	1.67 ± 1.33 (5)	1.16 ± 1.26 (16)	1.61 ± 1.35 (6)
28. Being seduced by someone	1.29 ± 1.26 (10)	0.88 ± 1.14 (10)	1.37 ± 1.25 (17)	1.33 ± .126 (7)	1.6 ± 1.29 (7)
1. Being in a romantic scenario (candlelit dinner, sunset walk, etc.)	1.27 ± 1.28 (11)	0.99 ± 1.25 (7)	1.52 ± 1.27 (12)	1.2 ± 1.22 (14)	1.32 ± 1.31 (16)
8. Masturbating a partner	1.27 ± 1.28 (12)	0.68 ± 1.07 (18)	1.6 ± 1.4 (8)	1.27 ± 1.15 (11)	1.51 ± 1.31 (11)
27. Seducing someone	1.27 ± 1.26 (13)	0.78 ± 1.07 (14)	1.38 ± 1.3 (16)	1.43 ± 1.33 (5)	1.58 ± 1.2 (9)
51. Remembering an erotic or pornographic scene from a movie, book, or comic	1.24 ± 1.26 (14)	1.08 ± 1.25 (4)	1.26 ± 1.27 (19)	1.2 ± 1.22 (13)	1.38 ± 1.27 (15)
9.Practicing oral sex (cunnilingus/fellatio/anilingus)	1.21 ± 1.29 (15)	0.62 ± 1.03 (22)	1.59 ± 1.34 (9)	1.2 ± 1.16 (15)	1.39 ± 1.31 (14)
25. Looking after a partner	1.2 ± 1.33 (16)	1.02 ± 1.23 (6)	1.43 ± 1.42 (14)	1.27 ± 1.32 (10)	1.11 ± 1.27 (19)
17. Using sex toys or other common objects for sexual purposes	1.17 ± 1.29 (17)	0.76 ± 1.1 (16)	1.39 ± 1.31 (15)	1 ± 1.21 (20)	1.39 ± 1.36 (13)
91. Engaging in sexual activity a known person (friend, colleague, etc.)	1.06 ± 1.17 (18)	0.63 ± 0.97 (20)	1.11 ± 1.18 (21)	1.1 ± 1.16 (19)	1.41 ± 1.21 (12)
89. Engaging in sexual activity with your regular partner	1.01 ± 1.32 (19)	0.39 ± 0.86 (40)	1.51 ± 1.5 (13)	0.96 ± 1.16 (21)	1.1 ± 1.3 (21)
4. Receiving a massage	1 ± 1.23 (20)	0.62 ± 1.02 (21)	1.28 ± 1.29 (18)	0.93 ± 1.14 (23)	1.11 ± 1.29 (20)
72. Being dominated/submissive	1 ± 1.27 (21)	0.78 ± 1.2 (15)	1.1 ± 1.32 (22)	1.12 ± 1.21 (18)	1.06 ± 1.27 (22)
53. Having sex with two people (threesome, ménage à trois)	0.96 ± 1.17 (22)	0.66 ± 1 (19)	0.93 ± 1.15 (28)	1.13 ± 1.17 (17)	1.24 ± 1.27 (18)
49. Remembering a past sexual experience	0.86 ± 1.14 (23)	0.33 ± .75 (48)	1.15 ± 1.22 (20)	0.94 ± 1.09 (22)	1.04 ± 1.21 (24)
11. Having a vaginal intercourse with an insertive role (top/active; with penis or sex toys)	0.83 ± 1.21 (24)	0.58 ± 1.05 (24)	1.05 ± 1.32 (23)	0.69 ± 1.04 (37)	0.89 ± 1.27 (30)
68. Being hit (spanked, slapped, whipped, etc.)	0.82 ± 1.18 (25)	0.52 ± 1.02 (28)	1 ± 1.25 (26)	0.78 ± 1.04 (30)	0.96 ± 1.24 (27)

## Discussion

The current study aimed to explore possible differences in the experience of sexual desire and fantasies among the groups belonging to the ACE spectrum, contributing to the growing evidence of heterogeneity within asexuality. As expected, different profiles emerged from the self-reported measures assessed, consistent with the definitions attributed to the groups (asexual, demisexual, gray-asexual, and questioning), although leaving room for individual variability.

The participants were predominantly young cisgender women and non-binary individuals assigned female at birth. This finding aligns with previous literature on asexuality (Bogaert, 2004; Brotto et al., 2010; Hermann et al., 2020; Weis et al., 2019), which showed that the most common gender identity among asexual communities was female (59%), with 13% identifying as male, and 27% reporting non-binary identities or questioning their gender identity. This gender disproportion has been interpreted as a result of societal gender norms, where having no/low sexual attraction is more socially accepted for women than men (Gupta, 2019). However, further research is needed to explore possible explanations for this gender gap. Consistent with the study by Copulsky and Hammack (2023), which highlighted a close link between sexual and romantic identification within the ACE spectrum, the asexual group was the least likely to be in a relationship and to experience romantic attraction toward one or more genders, while a more nuanced picture emerged for the other groups.

Regarding the frequency of sexual activity, the asexual group reported the lowest rates compared to the other groups (consistent with previous studies, e.g., Copulsky & Hammack, 2023; Hille et al., 2020). However, unexpectedly, no differences were found in terms of masturbation and porn consumption, with the majority of participants reporting engagement in solo sexual activities sometimes or often. This element prompts reflection on the distinction between participating in sexual activities involving one or more sexual partners (which appears to differentiate among the groups) and engaging in solo activities, which were common across the spectrum. This trend in sexual behavior is also reflected in the results concerning sexual desire and fantasies.

All the groups reported lower levels of desire compared to other studies that used SDI-2 and SDEF in the Italian allosexual women and men (Nimbi et al., 2023a). However, contrary to stereotypes (MacInnis & Hodson, 2012; Zivony & Reggev, 2023), most people in the ACE spectrum reported experiencing sexual desire. In terms of group differences in sexual desire domains (SDI-2 and SDEF1), the results appear to be consistent with the literature (Copulsky & Hammack, 2023; Hille et al., 2020), with asexual people reporting the lowest levels of desire, demisexual people reporting the highest, and gray-asexual people falling in the middle. Particularly noteworthy are the areas of Dyadic desire (SDI-2), sexual desire, regular partner, attractive partner, and responsive desire (SDEF1), which showed medium to large effect sizes. While Copulsky and Hammack (2023) found similar results when assessing sex drive and disposition toward engaging in sexual activity, the current results provide an important additional detail: The desire for partnered sexual activities has a greater effect in differentiating among the groups. This finding raises the possibility of considering the distinction between desire for others and a more self-centered desire, as speculated in relation to the behavioral aspects of masturbation and porn consumption. Moreover, it supports Bogaert’s important distinction (Bogaert, 2012a, 2015) between sexual desire and a lack of sexual attraction to others in the context of asexuality, such that asexual people are often best characterized as a lack of sexual attraction to others and not necessarily lacking in sexual desire.

Focusing on the frequency and use of erotic fantasies and discussing them with the results of other studies that used SDI-2 and SDEF in the allosexual population (Nimbi et al., 2023c), the scores in all domains for the four groups are lower, except for negative emotions and fantasies normality. Additionally, the asexual group showed lower scores than the other ACE groups. Interestingly, they also reported lower scores on negative emotions compared to the demisexual, gray-asexual, and questioning groups. In this sense, the data underscore how ACE people's experience of their desire is not negative or a source of possible distress. This element should sensitize sexual health clinicians even more to move away from a pathologizing view of asexuality, based on the stereotype that sexual attraction is something given to everyone.

Furthermore, questioning individuals scored higher than other groups in the negative feelings to sexual desire domain. This, along with their higher scores in negative emotions and the emphasis placed on sexual fantasies (SDEF2 subscales), may indicate a specific experience among questioning individuals. One possible explanation is that some questioning individuals may be within the process of defining one's identity within the ACE spectrum. As discussed for other LGBTQIA + identities (Levounis et al., 2012; Robbins et al., 2016), this process often involves phases of monitoring internal states, such as sexual desire, fantasies, and feelings of attraction. These internal states may serve as a reference point for self-identification and the formation of an identity (give oneself a name). The process of monitoring and rumination can also lead to experiencing negative emotions, especially when feelings are unclear, unresolved, and confused (Boyer & Lorenz, 2020). In this case, authors speculated that for some questioning individuals, it is possible that experiencing “excessive desire or too many fantasies” could be distressing, since it might fuel doubts about one's identity that move away from clichés on asexuality. Following the model of asexual coming out (Robbins et al., 2016), it is possible to speculate that some questioning participants may be in the process of accepting and negotiating the salience of their identity. They are aware of asexuality's existence, may have engaged with asexual communities, and are considering their place within the ACE spectrum. They are developing a more precise articulation of their identity that better describes their feelings while exploring alternative possibilities. This process is not necessarily distressing, but can become so, especially for individuals with a lower tolerance for uncertainty (Galupo et al., 2014).

Research has shown (Levounis et al., 2012; Luyckx et al., 2007) that while self-reflection and rumination play important roles in identity processing, for some individuals, the latter can hinder this process, making it challenging to explore and fully commit to their identities. Future investigation with respect to the experience of questioning people might be important to test this hypothesis.

Although research on the well-being of individuals who are uncertain about their sexual orientation is limited, it should be recognized that labels (e.g., asexual, demisexual, gay, etc.) can be an important tool in creating a sense of belonging to a specific community. While questioning individuals tend to share characteristics related to the way they experience sexual fantasies, they do not appear to have other specific patterns that differentiate them from the other three groups. It is possible to speculate that this is because the questioning group comprises individuals with diverse and fluid sexual attractions who currently struggle to label themselves and understand where they fit within the ACE spectrum (or if they fit anywhere at all).

Discussing the contents of sexual fantasies assessed with the SDEF3, the asexual group scored lower in all domains, with few exceptions (taboo area, sexual abuse, and dirty fetish) where no differences among the groups were found. Considering the mean scores of the allosexual population derived from the SDEF3 validation study (Nimbi et al., 2023b), all the groups scored lower in all fantasies contents domains, except for taboo and romantic fantasies, in which rates are similar to the ones reported by allosexual women. Looking at the most rated fantasies, it is possible to highlight that all the groups reported “caressing and hugging” and “kissing a partner” as the two most frequent fantasies. Here again, a different characterization of fantasies between the asexual, gray-asexual, and demisexual groups is repeated. Demisexual people are also more likely than the other groups to report fantasies that involve contextual and romantic scenarios with small to medium effect size. This is consistent with results from Hille et al. (2020), showing that, for demisexual participants, an emotional connection with partners is more likely to lead to sexual feelings of arousal than for asexual and gray-asexual people. Moreover, the demisexual group reported less often fantasies involving threesomes than the other groups, maybe because a scenario that involves more than one partner recalls a different emotional engagement, not in line with leading monogamous cultures.

Asexual individuals, on the other hand, reported more frequently engaging in scenarios that do not directly imply sexual intercourse, such as being in a romantic setting or taking care of a partner. They also reported creating sexual scenarios from memories, such as recalling an erotic or pornographic scene from a movie, book, or comic. In contrast, they rarely reported fantasies about regular partners or past sexual experiences compared to the other groups. Some participants have specified (in the space left open in the survey to add other information that the participant felt was relevant) that they are usually not the protagonists of their sexual fantasies (*n* = 29) and that they fantasize about faceless people (*n* = 34) or fictional characters (*n* = 8). This finding aligns with the existing literature (Bogaert, 2012a; Yule et al., 2017): Asexual sexuality appears to be activated by individuality rather than specific individuals, as discussed by Bogaert's (2012a) stating that some asexual individuals have an “identity-less sexuality” named autochorissexualism, characterized by a disconnection between their sense of self and a sexual object or target. It is important to emphasize that while research tends to assume that fantasizing about faceless people or scenarios that do not involve oneself is unique to asexual individuals (Bogaert, 2012a, b), there is no specific study that investigates the presence and consistency of this aspect in the allosexual population, making it impossible to make a comparison. It is possible that this kind of fantasies exists in the general population as well and may be more common than previously assumed. However, this aspect has not been explored yet, and therefore it may not necessarily be a peculiarity exclusive to asexual individuals. At the same time, it may be also reasonable to state that autochorissexualism very likely occurs at a higher frequency in asexual people relative to the fantasizing of allosexual people.

While results concerning asexual and demisexual individuals tend to show differences that are coherent with their definitions, gray-asexual participants showed less identifiable and more nuanced characteristics that put them in a real “gray area.” Further study is certainly needed in the future on the experience of gray-asexual people.

Although some limitations highlighted in previous studies on asexual individuals have been addressed, such as the use of reliable measures and adequately nurtured small samples, and a move away from relying solely on web-based asexual communities and forums, the results of the current study should still be interpreted with caution. Participants were recruited through social networks, which may have favored younger individuals who had access to the internet, smartphones, and/or computers, and possessed at least a minimum level of digital literacy. Moreover, the study advertisement was predominantly shared by sex-positive, LGBTQIA+, and feminist profiles, resulting in a specific group of participants that may not fully represent the diversity of individuals within the ACE spectrum.

The study relied on self-administered questionnaires, which can be susceptible to respondent bias and falsification. To mitigate this bias, a measure of social desirability was used as a covariate. However, it is important to acknowledge that self-report measures may still have limitations. In the context of studying complex areas such as sexuality and erotic fantasies, quantitative research can sometimes oversimplify and overlook nuances. Therefore, conducting future qualitative studies may be beneficial in capturing the complexity of sexual desire and the erotic imaginary experiences of individuals within the ACE spectrum. Furthermore, future research should aim to involve a more diverse range of participants, including older asexual individuals, men, and transgender individuals who were assigned male at birth, to explore intersectionalities among different identities within the ACE spectrum.

### Conclusions

This study showed some specific patterns of desire and fantasies among the asexual, gray-asexual, demisexual, and questioning groups, highlighting both differences and similarities in their experiences of low levels of sexual desire and fantasies. It is important to note that this study does not aim to provide a taxonomic description of sexual identities within the ACE spectrum. Instead, it aligns with a sex-positive approach that emphasizes individual self-determination and the fluidity of experiences and identities (Nimbi et al., 2021). However, understanding the heterogeneity and complexity of the ACE spectrum is relevant from a scientific and clinical perspective (Gupta, 2017; Pratt-Chapman et al., 2022; Schneckenburger et al., 2023).

One common bias that persists today is the perception of asexuality as a complete absence of any form of sexual thought, fantasy, and behavior or as being hostile toward sexuality (MacInnis & Hodson, 2012; Zivony & Reggev, 2023). This study offers a more realistic view of asexuality and challenges these misconceptions, also providing valuable data for clinicians working with asexual patients. Many asexual individuals report negative experiences with mental health providers due to biases and a lack of knowledge about asexuality (Herbitter et al., 2021). While guidelines for clinicians who work with asexual patients emphasize the need to deconstruct assumptions about sexuality being a necessary part of human life (Ginicola et al., 2017; Gupta, 2017; Pratt-Chapman et al., 2022; Schneckenburger et al., 2023), it is also important to recognize and discuss the diversity and heterogeneity within the ACE spectrum, a topic that is rarely addressed in the literature (Jones et al., 2017). Clinicians need to approach their ACE spectrum patients with an open mind, sensitivity, and an understanding of the diverse range of experiences and desires within this spectrum. Clinicians should not assume that their ACE spectrum patients are completely disinterested in sex, both solitary and partnered. Additionally, it is crucial to recognize that some individuals within the ACE spectrum may have a strong interest in cultivating the sexual aspect of their intimate relationships.

## Data Availability

Data are available on request to the corresponding author.
